# Beyond Primary HER2 Expression: Trastuzumab Deruxtecan’s Efficacy in Brain Metastasis

**DOI:** 10.3390/cancers16203525

**Published:** 2024-10-18

**Authors:** Glori Das, Stephen T. C. Wong, Hong Zhao

**Affiliations:** 1 Department of Systems Medicine and Bioengineering, Houston Methodist Neal Cancer Center, Houston Methodist Hospital, Weill Cornell Medical College, Houston, TX 77030, USA; 2 Department of Biomedical Engineering, Texas A&M University School of Medicine, College Station, TX 77843, USA

## Abstract

This commentary focuses on the DESTINY-Breast12 study, published in Nature Medicine on 13 September 2024, which evaluates the efficacy of Trastuzumab deruxtecan (T-DXd) in treating HER2-positive advanced breast cancer, including those with brain metastases. We emphasize the broadened clinical potential of T-DXd in treating brain metastases from tumors originally classified as HER2-null or HER2-low, extending beyond its current use for breast cancer. This expanded application of T-DXd could provide new hope to patients dealing with challenging brain metastases, addressing an urgent need for effective treatment options.

## 1. Summary of DESTINY-Breast12 Study on T-DXd for HER2-Positive Brain Metastases

Trastuzumab deruxtecan (T-DXd) is a next-generation antibody–drug conjugate (ADC) that has received accelerated FDA approval for HER2-expressing, unresectable, or metastatic solid tumors. In a recent study published in Nature Medicine, Harbeck and colleagues presented findings from the DESTINY-Breast12 study, which evaluated T-DXd in treating patients with HER2-positive breast cancer with brain metastases.

Patients with metastatic disease, particularly those with brain metastasis, have poor outcomes and limited therapeutic options. The DESTINY-Breast12 study—the largest prospective trial to date—demonstrates the efficacy of T-DXd in treating HER2-positive brain metastases. The study reported a 12-month central nervous system (CNS)-specific progression-free survival (PFS) rate of 58.9% (95% CI: 51.9–65.3) and an objective response rate (ORR) of 71.7% (95% CI: 64.2–79.3) [1]. This is particularly significant when contrasted with prior HER2-targeted therapies, which have shown variable efficacy due to poor penetration of the blood–brain barrier [2,3,4], including HER2-directed antibodies such as trastuzumab and pertuzumab, antibody–drug conjugates like T-DM1, and small-molecule tyrosine kinase inhibitors such as lapatinib and tucatinib. The substantial intracranial response to T-DXd highlights its unique capacity to overcome this critical barrier in controlling HER2-positive brain metastases.

## 2. Broadening Perspectives on T-DXd’s Therapeutic Potential

HER2-positive tumors in the DESTINY-Breast12 study were classified based on primary tumor HER2 scores, as is typical in clinical trials. However, it is increasingly recognized that HER2 expression can differ between primary and metastatic tumors, including brain metastases, due to tumor heterogeneity and evolution. Studies indicate a discordance rate of up to 12% in HER2 status between primary breast tumors and brain metastases [5,6], with evidence suggesting HER2 upregulation in brain metastases even when primary tumors are HER2-null or HER2-low. For instance, studies on matched breast tumor and brain metastasis specimens have shown that as many as 50% of HER2-null primary tumors correspond with HER2-positive brain metastases [5,7]. Moreover, circulating tumor cells (CTCs) from patients with HER2-null primary tumors have been found to express HER2 once metastasized to the brain [8]. Given these discrepancies, direct assessment of HER2 in brain metastases is not always feasible. However, evidence strongly supports the clinical utility of T-DXd in treating patients with brain metastases, even those without HER2-positive primary tumors.

The DEBBRAH trial’s findings further substantiate this view, with intracranial ORRs of 50% in untreated asymptomatic brain metastases and 33.3% in progressing brain metastases in patients with HER2-low breast cancer [9]. Additionally, the DESTINY-Breast06 and DAISY II studies demonstrate that T-DXd provides clinical benefit even in HER2-low and HER2-ultralow metastatic breast cancer, reducing the risk of disease progression significantly [10,11]. These data suggest that T-DXd could be an effective treatment for brain metastases in HER2-null, low, or ultralow tumors.

These findings also raise an intriguing question: Does T-DXd’s efficacy operate independently of HER2 binding, or would more sensitive measures of HER2 expression further stratify responses [12]?

Beyond breast cancer, T-DXd has become a new standard of care for patients with treatment-refractory HER2-positive gastrointestinal cancers, HER2-mutant non-small-cell lung cancer, and other HER2-overexpressing solid tumors owing to its potent anti-tumor activity and impressive clinical efficacy. In the DESTINY-Lung01 trial’s HER2-overexpressing cohort, 29 of 90 patients (32%) had stable or treated brain metastases and showed outcomes similar to the overall intention-to-treat population [13]. HER2 upregulation in brain metastasis has been reported in gastrointestinal cancers [14], and the DESTINY-Gastric01 trial suggested a potential benefit in patients with HER2-low gastric cancer [15]. This broad applicability suggests that T-DXd could also be a valuable option for treating brain metastases in these populations, extending its clinical impact beyond traditional HER2-positive breast cancer.

Furthermore, the high objective response rates achieved with T-DXd in brain metastases and extracranial lesions indicate its potential value for patients with active leptomeningeal disease, a condition in desperate need of effective therapies.

## 3. Conclusions

The DESTINY-Breast12 study marks a significant milestone in the treatment of HER2-positive breast cancer with brain metastases, establishing T-DXd as a powerful therapeutic agent capable of overcoming the challenges posed by the blood–brain barrier—a limitation often encountered with previous HER2-targeted therapies. The remarkable efficacy of T-DXd, as evidenced by its CNS-specific progression-free survival and objective response rates, offers new hope to patients who previously had limited options. Moreover, the study illuminates the potential for T-DXd to benefit a wider array of patients, including those with varying levels of HER2 expression and even those with HER2-negative primary tumors that express HER2 upon brain metastasis. This adaptability of T-DXd to target a broad spectrum of HER2 statuses, along with its demonstrated efficacy in other solid tumors, suggests its role as a cornerstone treatment in oncology. Future studies should continue to explore the full potential of T-DXd, particularly in diverse tumor contexts and in combination with other therapies, to fully harness its therapeutic promise for patients suffering from devastating brain metastatic cancers.
